# Some Observations on Diseases of the Digestive Organs

**Published:** 1906-09-08

**Authors:** 


### Hospital Clinics.

### SOME OBSERVATIONS ON DISEASES OF THE DIGESTIVE ORGANS.

### DYSPEPSIA AND THE NERVOUS SYSTEM.

#### General Observations.

As illustrating the relationship between dyspepsia and the nervous system, so much insisted on by Hutchison, may be quoted some interesting observations on a series of 22 melancholics in an asylum in America by Cowie and Inch.[1] Their results, which closely correspond to some earlier observations by van Noorden, show that true hyperchlorhydria with increased peptic power and motor power are fairly common among melancholic lunatics, both sexes being equally affected. Taking the two series together, it was found in 77.7 per cent. (38 cases). They attribute this to purely nervous causes, as increased secretion was in some cases associated with definite and widespread degenerative changes in the glands and mucous membrane of the stomach. They also found that organic disease, inflammatory conditions, and painful neuroses were frequent in the insane, and are of opinion that adequate treatment of these conditions is likely to ameliorate the mental condition of the patient. The importance of "psychical" treatment in cases of "atony" or motor insufficiency is also brought out strongly by Kaufmann.[2] Excluding cases of grave insufficiency and severe inflammatory conditions, he points out that in doubtful cases of ulcer it is better at first to assume its absence. Apart from bad cases of neurasthenia and hysteria, a good prognosis as to improvement and as to life may be given. The patients are always mentally depressed, and need encouragement and stimulation.

As to diet, while insisting on its importance to a greater extent than Hutchison, Kaufmann practically bases his advice on this point on very similar grounds. He advises a more liberal diet, with weekly weighings to show the patient that he is improving. Food, according to Kaufmann, should be given often, in small quantities and in fairly concentrated form. Fluids should be restricted, and milk has been overvalued in this condition. Cream, however, may be ordered to the amount of 500 c.c., or about $\frac{7}{8}$ of a pint. If there is much hyperacidity meat should be only taken in small quantities and only at two meals. Irritating food should be avoided, vegetables are allowed, and even potato in the form of purée. Surmount,[3] on the other hand, in cases of hyperchlorhydria, accompanied by symptoms of dyspepsia, considers diet to be of great importance. Spices, condiments, fatty foods, alcohol, game, and most fruits and vegetables are to be avoided, and a bland albuminous diet prescribed; milk, custard, lean meat, chicken, toast are given frequently in small amounts, the meals gradually to be reduced in number and increased in amount.

#### Organic Lesions of the Stomach.

Pyloric stenosis may be congenital or acquired, the latter being generally a sequence of ulcer of the pylorus. Quintard[4] mentions instances in which the pyloric obstruction is not due to this cause, but merely to inflammatory swelling of the mucosa, leading temporarily to stenosis. Rest in bed with lavage and rectal feeding for a few days sets the condition to rights, but it is always liable to recur and produces uncontrollable vomiting. The diagnosis in all these conditions is made by a thorough examination of the stomach-contents and an estimation of the size of the organ. Einhorn[5] draws attention to the fact that continuous hypersecretion of a neurotic type is a clinical entity, and the exact diagnosis has a very important bearing on treatment, as only when an organic lesion exists is any benefit likely to accrue from surgical measures. With regard to ulcer, Thompson,[6] while not prepared to accept the classification proposed by West and others into acute and chronic cases, the former characterised by a single large hæmorrhage, and the latter by numerous slight ones, discusses the difficulty of drawing any conclusions from the records of hospitals where so-called "cured" or "relieved" cases are lost sight of as soon as they are discharged. He points out, however, the curious fact that cases of hæmatemesis diagnosed as ulcer are especially common among girls of the servant and lower middle classes, and are seldom seen among the labouring and lower classes. He quotes cases illustrating diagnostic difficulties, in some of which, without any symptoms, except dilatation, old gastric ulcer was found; and others in which, after profuse hæmatemesis, merely a congestion of the mucous membrane was found post-mortem. These latter cases are well recognised in this country, and have been fully studied by Hale White and Dawson Williams. Dowd[7] calls attention to the increase in deaths from cancer in the United States. The census for 1900 shows that there were during that year at least 9,000 deaths from cancer of the stomach, or 65 per cent. more than from appendicitis. As to treatment, he insists that surgical operation in the early stages is the only possible procedure, and that the fatality of the operation is not greater than that of many others more frequently performed. Vomiting he considers not to be a constant or early symptom; it had lasted seven months before admission to hospital in 73 per cent. of his cases. The vomiting is also variable in occurrence; sometimes constant, at other times irregular. In doubtful cases he advises a small exploratory incision. Dilatation of the stomach may occur as a result of a bulky diet, and Suzuki,[8] a distinguished Japanese naval surgeon, states that it is very common among the poorer classes in Japan, whose food consists largely of barley, rice, potatoes, and vegetables. He states that he has seen many post-mortems in people of this class in which the stomach was three or four times the size of the normal European or American stomach. It would be interesting to know whether a similar condition obtains among the poorer Irish peasantry.

### Gastric Functions in Old People.

It is a matter of common experience that old people generally require less food and have less power of digestion and assimilation than those in the prime of life. Gallerani,[9] in face of considerable practical difficulties, has made observations on 12 persons all over 70 and all free from digestive disturbances or any other condition of ill-health. He found that HCl was generally diminished, sometimes normal, never abolished; that it bore no relationship to pepsin secretion, which was very variable and sometimes high. The motility was usually impaired, and in many cases was diminished to almost a pathological extent; an hour after a test breakfast as much as 246 c.c. was sometimes found in the stomach, and iodopin gave the iodine reaction in the saliva before the end of an hour in two cases only. As might be expected, therefore, on *à priori* grounds, loss of muscular power is the main feature in the stomachs of the aged.

### Drugs.

Drugs, intelligently used, are of considerable value, but they must be employed with a definite purpose. Of pepsin Hutchison says little good, and this corresponds with the recently expressed opinion of Bettmann,[10] who says it is rarely of use in chronic cases of dyspepsia or in organic disease. In a few acute temporary conditions it may be of service, and should then be given in adequate doses—namely, 2 ounces of a .35 per cent. solution in 1 per cent. HCl. Bettmann, by the way, denies that the administration of artificial gastric juice impairs the secreting power of the stomach. The value of alkalies to correct hyperacidity is a somewhat debated point. Hutchison recommends the employment of earthy carbonates, such as mag. carb. after meals to neutralise excessive secretion, and thinks that soluble carbonates of the alkalies, such as sodii bicarb., before meals may be given to increase the secretion of gastric juice. Surmont coincides with this view, and gives calcium carbonate after meals in cases of hyperchlorhydria, and mag. carb. if, in addition, constipation is present. Bismuth and the ordinary gastric sedatives are recommended by Hutchison for relief of hyperæsthesia when this is present; Surmont directs that bismuth should be administered in the morning in the form of a milk. All writers seem agreed that belladonna is of little or no value in practice for diminishing the amount of gastric secretion, with the exception of Elsner,[11] who states that belladonna and supra-renal extract are of value in cases of hypersecretion, where there is either organic or functional stenosis of the pylorus.

### Examination of Stomach-Contents.

The importance of an accurate determination of the stomach-contents is so great that Boas's [12] paper on common errors in carrying out the details of the procedure is very opportune. In the first place, it is important that before the test meal is given the stomach should be empty, and this can only be ascertained by a preliminary use of the tube. Then the meal must be of a definite constitution. Ewald's meal consists of 35 grams of white bread and 400 grams of water or tea without milk or sugar. Deviations from this lead to results which are not strictly comparable. Then it is of great importance that the stomach-contents should be removed at the proper time—namely, one hour after the meal. Owing to the variability in the amount of HCl which occurs in some individuals, the test should always be repeated when wide deviations from the normal are found. Peptic and milk-curdling powder should also be tested, but it is only in the absence of blood, bile, mucus, and saliva that accurate quantitative results can be obtained. The tests for free acid (HCl) are also frequently made in a manner which is totally unreliable. Whitney [13] calls attention to Hoffmann's method, which shows not merely the actual amount of free acid, but the amount capable of exerting its action by means of free hydrogen ions. Free hydrogen ions saponify methyl acetate with the formation of the corresponding alcohol and acid; the latter can be titrated with caustic potash. The procedure is fairly simple, and consists of keeping four flasks at a temperature between 50° and 55° C. for at least four hours. The first flask contains 2 c.c. methyl acetate and 10 c.c. gastric juice; the second, the same with 10 c.c. $\frac{N}{40}$ HCl; the third, 10 c.c. gastric juice alone; and the fourth, 2 c.c. methyl acetate and 10 c.c distilled water. Thus, after titration of each flask we know (1) the total acidity (free and combined HCl), (2) the increase due to formation of acetic acid, (3) any acidity due to saponifying action of water. By subtracting the last two from the first we obtained the value of the acetic acid liberated in 10 c.c. gastric juice in terms of $\frac{N}{10}$ KOH; we can also determine from the second tube the value in terms of $\frac{N}{10}$ KOH of the acetic acid in 10 c.c. $\frac{N}{40}$ HCl. One divided by the other, after correction for saponifying action of water, and the result divided by .091, gives the percentage of free acid in the gastric contents. An improved method of obtaining the gastric contents has been suggested by Turck,[14] who uses a double tube. Into one tube air is blown into the stomach, while the contents are forced out by the other. This obviates the possibility of nipping up the mucous membrane or getting the tube blocked by particles of solid food.

[1] Amer. Jour. Med. Sci., Sept. 1905, p. 460. [2] Brit. Med. Jour. Epit., Jan. 13, 1906, No. 18. [3] Brit. Med. Jour. Epit., Dec. 9, 1905, No. 358. [4] Med. Rec., Jan. 13, 1906, p. 79. [5] Med. Rec., Jan. 13, 1906, p. 79. [6] Amer. Jour. Med. Sci., Sept. 1905, p. 375. [7] Med. Rec., Jan. 20, 1906, p. 91. [8] Med. Rec., Jan. 13, 1906, p. 79. [9] Brit Med. Jour, Epit., Oct. 21, 1905, No. 246. [10] Edin. Med. Jour., Dec. 1905, p. 562. [11] Med. Chron., Feb. 1906, p. 307. [12] Brit. Med. Jour. Epit., Jan. 6, 1906, No. 1. [13] Med. Chron., Feb. 1906, p. 305 [14] Ibid., p. 306.

